# Childhood trauma and cardiometabolic risk in severe mental disorders: The mediating role of cognitive control

**DOI:** 10.1192/j.eurpsy.2021.14

**Published:** 2021-03-29

**Authors:** Synve Hoffart Lunding, Carmen Simonsen, Monica Aas, Linn Rødevand, Maren Caroline Frogner Werner, Jannicke Fjæra Laskemoen, Gabriela Hjell, Petter Andreas Ringen, Trine Vik Lagerberg, Ingrid Melle, Ole A. Andreassen, Torill Ueland, Nils Eiel Steen

**Affiliations:** 1 NORMENT, Division of Mental Health and Addiction, Oslo University Hospital & Institute of Clinical Medicine, University of Oslo, Oslo, Norway; 2 Early Intervention in Psychosis Advisory Unit for South East Norway, Oslo University Hospital, Oslo, Norway; 3 Department of Psychiatry, Ostfold Hospital, Graalum, Norway; 4 Division of Mental Health and Addiction, Oslo University Hospital, Oslo, Norway; 5 Institute of Clinical Medicine, University of Oslo, Oslo, Norway; 6 Department of Psychology, University of Oslo, Oslo, Norway

**Keywords:** Adverse childhood experiences, bipolar and related disorders, cognition, schizophrenia spectrum and other psychotic disorders, waist circumference

## Abstract

**Background:**

Cardiometabolic risk is increased in severe mental disorders (SMDs), and there appears to be a relationship between childhood trauma and cardiometabolic risk, possibly related to adverse health behavior. The current study examined the association between childhood trauma and serum lipids and adiposity in SMDs and the potential mediating role of cognitive and personality characteristics.

**Methods:**

Participants with schizophrenia and bipolar spectrum disorders (*N* = 819) were included, cardiometabolic risk factors (*serum lipids*, *body mass index*, and *waist circumference)* were measured, and history of *childhood trauma* was assessed by the Childhood Trauma Questionnaire. Cognitive and personality characteristics were available in subsamples, with assessments of cognitive control, impulsiveness, self-esteem, and affective lability. Linear regressions and mediation analyses with Hayes’ PROCESS were performed, adjusting for age, sex, antipsychotic agent propensity of metabolic side-effect, and diagnostic group.

**Results:**

Experience of three or more subtypes of childhood trauma was positively associated with waist circumference in patients with SMDs (*p* = 0.014). There were no other significant associations between trauma variables and lipid or adiposity measures in the total sample. Cognitive control was a significant mediator between experience of one or two subtypes of childhood trauma and waist circumference.

**Conclusions:**

The results indicate childhood trauma as a predisposing factor for increased waist circumference in individuals with SMDs. Poorer cognitive control, suggestive of adverse health behavior, might be a mediating factor of the association, and the findings indicate the potential importance of increased focus on these factors in prevention and treatment regimens targeting cardiometabolic health.

## Introduction

Schizophrenia and bipolar disorder are severe mental disorders (SMDs) with lifetime prevalences of approximately 1% [1,2]. They have overlapping clinical features and genetic susceptibility [3] and are among the most disabling and costly disorders worldwide [4]. Compared to the general population, life expectancy for people with SMDs is reduced by about 15 years [5,6] and the mortality rates are close to doubled, with both natural and unnatural causes of death being of significance [7–9].

Cardiovascular disease (CVD) is one of the leading causes of the excessive mortality in SMDs [10,11]. The development of CVD is based on complex, multifactorial mechanisms. CVD risk in SMDs may partly be related to an inherent genetic susceptibility [12–14], but appears mainly to be driven by environmental factors, including lifestyle (unhealthy diet and physical inactivity), smoking, and metabolic side-effects of medication [15,16]. Moreover, recent findings indicate a genetic architecture of schizophrenia protective against weight gain [17], highlighting the role of medication and behavioral mechanisms in weight gain for this group [18].

Childhood trauma is a well-recognized risk factor for psychiatric disease [19] and is associated with cardiometabolic risk in the general population [20]. A meta-analysis of 41 studies of varying samples reported an odds ratio of 1.36 (95% confidence intervals [CI]: 1.26–1.47) for developing obesity over the life course due to childhood maltreatment [21]. Similarly, early-life traumatic events have been associated with metabolic dysregulation in SMDs [22–26], although data across different disorders and cardiometabolic risk factors are sparse. Nevertheless, in a previous case–control study of childhood abuse, body mass index (BMI), and inflammatory markers in a smaller, partly overlapping sample of the current study, Aas et al. [27] found that participants with SMDs and healthy controls reporting more types of childhood abuse were more likely to have high BMI, consistent with a few other reports of SMDs [22,23].

Previous research also suggests that childhood trauma is associated with adverse health behavior [28]. Still, the psychological mechanisms underlying the relationship between childhood adversity and behavior-related cardiometabolic risks are largely unknown. Eating triggered by emotions is a possible mechanism of the relationship [29]. Emotional eating can be described as an overconsumption of food as a reaction to negative emotions and is associated with increased weight [30]. Eating behavior including emotional eating has, in non-psychiatric studies, been associated with personality characteristics of impulsiveness [30–32] and emotional dysregulation [33,34], features also linked to childhood adversity [35–38]. A similar relationship of personality characteristics and eating behavior is found in SMDs [39–41]. Furthermore, recent studies of SMDs indicate a relationship between cognitive functioning and BMI or waist circumference [42–45]. Impaired cognitive function also appears to be related to childhood trauma in the same disorders [46,47]. Moreover, affective components, such as affective lability and affective intensity, have been linked to early trauma [48] as well as to elevated BMI in SMDs [49]. To our knowledge, there are only a few earlier studies, but none specific for SMD, providing simultaneous data on childhood experiences, cognitive and personality characteristics, and metabolic factors [50,51], enabling investigation of the potential mediating role of psychological features.

In the current study, we investigate the relationship between childhood trauma and different weight-related cardiometabolic risk factors including serum lipids in a large sample of individuals with SMDs. Furthermore, we examine the potentially mediating role of cognitive and personality characteristics on the relationship between childhood trauma and cardiometabolic risk factors. We hypothesize a positive association between childhood trauma and cardiometabolic risk factors mediated by cognitive control, impulsiveness, self-esteem, and affective lability.

## Methods

### Organization and recruitment

The current study was part of the Thematically Organized Psychosis (TOP) Study, an ongoing multicenter study at the NORMENT Centre for Psychosis Research, in Oslo, Norway. Inpatients and outpatients from psychiatric hospital units in the Oslo area were recruited. General inclusion criteria for all participants were aged 18–65 years, meeting the criteria in Diagnostic and Statistical Manual of Mental Disorders, 4th edition [52] for schizophrenia spectrum (SCZ) or bipolar spectrum (BD) disorders, speaking, and understanding a Scandinavian language sufficiently well for valid assessments, and being able and willing to give written, informed consent. Exclusion criteria were as follows: history of severe head injury, severe somatic illness, neurological disorder, or a marked cognitive deficit (IQ < 70).

A total of 819 participants included from 2006 to 2017 completed the Childhood Trauma Questionnaire (CTQ) [53–55], including 459 participants with SCZ (schizophrenia, schizophreniform disorder, schizoaffective disorder, delusional disorder, and psychotic disorder not otherwise specified [NOS]) and 360 participants with BD (bipolar disorder I, bipolar disorder II, bipolar disorder NOS, and Major Depressive Disorder with Psychotic Features).

The study was conducted in line with the Declaration of Helsinki and approved by the Regional Committee for Medical Research Ethics as well as the Norwegian Data Inspectorate. All participants signed written, informed consent.

### Clinical and cognitive assessment

Diagnostic interviews were performed by trained physicians and psychologists using The Structured Clinical Interview for DSM-IV Axis I Disorders (SCID-I) [56]. All diagnostic raters received regular clinical supervision from senior researchers and professors, both individually and in groups. Inter-rater reliability was assured by scoring a series of videos [57]. A good inter-rater reliability for diagnostic assessments at the TOP study was indicated, with an overall kappa score between 0.92 and 0.99 across assessment teams.

The degree of current *psychotic symptoms* was measured with The Positive and Negative Syndrome Scale (PANSS) [58], current *depressive symptoms* with Inventory of Depressive Symptomatology-Clinician Rated [59], and current *manic symptoms* with the Young Mania Rating Scale [60]. *Cognitive control* was measured by the inhibition condition from the Color–Word Interference Test, Delis–Kaplan Executive Function System [61], and *impulsiveness* with the Barratt Impulsiveness Scale (BIS-11) [62]. *Self-esteem* was measured by the Rosenberg Self-Esteem Scale (RSES) [63] and for *affective lability* we used a Norwegian short form of the Affective Lability Scale (ALS-18) [64–66]. For Cognitive control, the total time to complete the test was reported (i.e., raw scores) with higher scores representing poorer cognitive functioning. For ALS-18, the total score of affective lability is the sum of all item responses divided by 18. *Intellectual functioning* was assessed using the Wechsler Abbreviated Scale of Intelligence (WASI), two-subtest version [67].

### Childhood Trauma Questionnaire

A Norwegian 28-item version of the CTQ was used in the current study [53–55]. CTQ is a retrospective questionnaire assessing traumatic experiences in childhood according to five categories of trauma; physical, emotional, and sexual abuse, and physical and emotional neglect. Each category is scored on a five-point Likert-type scale ranged from “never true,” “rarely true,” “sometimes true,” “often true,” to “very true.” The score for each trauma category range between 5 and 25 with a higher score indicating more severe maltreatment. For each trauma category, experience of childhood trauma was defined as reaching a moderate to severe cutoff score [68].

Based on the five trauma categories and the moderate to severe cutoff scores, we created the variables no subtypes of trauma (not reaching the moderate to severe cutoff score for any of the trauma categories), one or two subtypes of trauma (reaching the moderate to severe cutoff score for one or two trauma categories), and three or more subtypes of trauma (reaching the moderate to severe cutoff score for three or more trauma categories). Based on the literature, we also investigated separately the abuse dimension of CTQ (emotional abuse, physical abuse, and sexual abuse) using the following categorization: no subtypes, one subtype, two subtypes, or three subtypes of abuse [27]. For the descriptive analyses, the five scores representing the different categories of trauma were summed up to yield a total score.

### Biochemical assessments

Blood samples, including total cholesterol (TC), high-density lipoprotein cholesterol (HDL-C), low-density lipoprotein cholesterol (LDL-C), and triglycerides (TGs) in serum were routinely collected according to the TOP protocol and analyzed at the Department of Medical Biochemistry, Oslo University Hospital by standard methods. Biological sampling procedures were done within 2 weeks of the symptom assessments.

### Physical assessment and psychopharmacological treatment

Patients had their weight, height, and waist circumference measured by a trained physician or nurse. Weight was measured while wearing light clothing. Based on their weight and height, BMI was calculated using body mass weight in kilograms divided by the square of the height in meters (kg/m^2^) according to the World Health Organization [69]. Waist circumference was measured midway between the lowest rib and the iliac crest.

Current and the last 5 years of treatment with psychopharmacological agents were recorded based on clinical interviews and medical records.

### Statistical analyses

Analyses were performed using IBM SPSS Statistics for Windows, Version 25.0 [70]. The distribution of data was assessed by Kolmogorov–Smirnov tests, histograms, and Q–Q plots. Dependent variables with non-normal distributions were log transformed before entered into statistical analyses. Descriptive analyses were performed comparing sociodemographic and clinical variables between SCZ and BD. For continuous variables with a normal distribution, independent samples *t*-tests were used. Mann–Whitney *U* test was applied for data with non-normal distribution. When comparing the proportion of males versus females in each study group, chi-square test for categorical variables was used.

#### Total effect analyses

To investigate the relationship between childhood trauma and the range of lipid and adiposity measures (TC, HDL-C, LDL-C, TGs, BMI, and waist circumference), we conducted linear regression analyses; lipid and adiposity measures were set as dependent variables, and number of childhood trauma subtypes (none vs. one or two subtypes vs. three or more subtypes), age, sex, diagnostic group (SCZ and BD), and antipsychotic agent (AP) propensity of metabolic side-effect (no AP, low, and high) based on current AP use were set as independent variables. Propensity of metabolic side effect of APs was categorized according to De Hert et al. [71]. Findings of significant trauma effects were additionally tested in a separate model by adjusting for AP propensity of metabolic side-effect based on AP use previous 5 years. By including SCZ and BD as predictors, differences in lifestyle and health risk behavior associated with diagnoses were controlled for. Subanalyses were performed for the SCZ and BD groups separately.

#### Mediation effect analyses

Data on cognitive and personality characteristics were available in subsets of the sample (cognitive control, *N* = 677 [SCZ *N* = 363; BD *N* = 314]; BIS-11, *N* = 323 [SCZ *N* = 173; BD *N* = 150]; RSES, *N* = 632 [SCZ *N* = 366; BD *N* = 266]; ALS-18, *N* = 196 [SCZ *N* = 72; BD *N* = 124]). Mediation analyses were performed in these subsets to investigate if identified relationships between childhood trauma and lipids and adiposity in the total effect analyses of the complete sample could be mediated by these cognitive and personality characteristics. For the mediation analyses, we applied Hayes’ [72] regression based approach using PROCESS, version 3.4 for SPSS. We tested cognitive control, impulsiveness, self-esteem, and affective lability as mediators in separate analyses, adjusted for the same variables as in the total effect analyses. In order to analyze the three partitioned independent variable, number of childhood trauma subtypes, we used a procedure described by Hayes [72] based on indicator coding, with the group coded with the smallest number set as reference category. Testing of the indirect effect was based on a bootstrap estimation approach with 5000 samples. Level of significance was adjusted to *p* ≤ 0.025 (0.05/2) based on testing of adiposity and lipid measures. Mediation was determined significant based on 95% confidence intervals not including zero [72].

## Results

### Sample characteristics

Sociodemographic, clinical, and cognitive characteristics of the sample (*N* = 819) are summarized in Table 1. Individuals in the SCZ group were significantly younger and there were more men in the SCZ group (both *p* < 0.001) as compared to the BD group. The SCZ group had significantly higher scores on cognitive control, whereas the BD group had significantly higher scores on IQ (both *p* < 0.001). The SCZ group had significantly more symptoms as measured by PANSS (*p* < 0.001)*.* Lipids differed significantly across groups (*p* values from 0.001 to 0.032) and the SCZ group had significantly higher waist circumference (*p* = 0.039) than the BD group.

**Table 1.** Sample characteristics according to study group.

	SCZ (*N* = 459)	BD (*N* = 360)	*p* value^g^
	*N* (%)	*N* (%)	
Sex, male	276 (60.1)	155 (43.1)	<**0.001**
Childhood trauma^a^	234 (51.0)	179 (49.7)	0.774
1 or 2 subtypes of trauma	152 (33.1)	112 (31.1)	0.593
≥3 subtypes of trauma	82 (17.9)	67 (18.6)	0.854
Above cutoff for moderate depression^b^	192 (42.7)	110 (32.3)	0.505
	*Median (IQR)*	*Median (IQR)*	
Age	26.0 (12.0)	30.0 (18.0)	<**0.001**
Age, first treatment psychosis^c^	24.0 (8.0)	–	
Age, first treatment mania^d^	–	26.0 (16.0)	
YMRS^e^	–	2.0 (4.0)	
IDS-C^e^	–	15.0 (17.0)	
CDSS^e^	5.0 (7.0)	–	
Childhood trauma^e^	40.0 (19.0)	39.0 (19.0)	0.632
Cognitive control^f^	58.0 (21.0)	53.5 (18.3)	<**0.001**
Body Mass Index (kg/m^2^)	25.2 (5.5)	25.5 (7.5)	0.418
Triglycerides (mmol/L)	1.2 (1.0)	1.1 (0.8)	**0.032**
	*Mean (SD)*	*Mean (SD)*	
IQ	99.6 (15.6)	107.9 (12.7)	<**0.001**
PANSS^e^	62.7 (16.3)	45.7 (10.3)	<**0.001**
Waist circumference (cm)	90.8 (15.1)	88.6 (13.3)	**0.039**
Total Cholesterol (mmol/L)	5.0 (1.0)	4.9 (1.0)	**0.016**
HDL-Cholesterol (mmol/L)	1.3 (0.5)	1.4 (0.6)	**0.001**
LDL-Cholesterol (mmol/L)	3.1 (1.3)	2.9 (1.1)	**0.001**
Impulsiveness^e^^,^^f^	67.4 (10.7)	69.4 (11.6)	0.117
Affective lability^e^^,^^f^	1.12 (0.08)	1.17 (0.07)	0.619
Self-esteem^e^^,^^f^	24.6 (6.4)	25.4 (6.7)	0.143

Missingness: Body Mass Index, 6.3%; Waist circumference, 9.3%; Lipids, 17.2–18.4%; Age of first drug treatment for psychosis, 16.6%; Age of first drug treatment for mania, 31.0%

Abbreviations: Affective lability, Affective Lability Scale (ALS-18); BD, Bipolar Spectrum Disorders (Bipolar I, Bipolar II, Bipolar Not Otherwise Specified, Major Depressive Disorder with Psychotic Features); CDSS, Calgary Depression Scale for Schizophrenia; Childhood Trauma, Childhood Trauma Questionnaire (CTQ); Cognitive control, The Inhibition condition in the Color–Word Interference Test, Delis–Kaplan Executive Functioning System (D-KEFS); HDL-Cholesterol, High-density lipoprotein-Cholesterol; IDS-C, Inventory of Depressive Symptomatology, Clinician-Rated; Impulsiveness, Barratt Impulsiveness Scale (BIS-11); IQ, Intelligence Quotient, based on Wechsler Abbreviated Scale of Intelligence (WASI), two-subtest version; IQR, interquartile range; LDL-Cholesterol, Low-density lipoprotein-Cholesterol; PANSS, Positive and Negative Syndrome Scale; SCZ, Schizophrenia Spectrum Disorders (Schizophrenia, Schizophreniform, Schizoaffective, Other psychosis); Self-esteem, Rosenberg Self Esteem Scale (RSES); YMRS, Young Mania Rating Scale.

a Meeting the moderate to severe cutoff score for ≥1 subtype(s) of childhood trauma.

b CDSS cutoff for moderate depression ≥ 6; IDS-C cutoff for moderate depression ≥ 22.

c Age of first drug treatment for psychosis in SCZ.

d Age of first drug treatment for mania in Bipolar I disorder.

e Total score (standardized scores for affective lability).

f Subsamples: cognitive control, *N* = 677; impulsiveness, *N* = 323; affective lability, *N* = 196; self-esteem, *N* = 632.

g Mann–Whitney *U* Test for variables represented by median (IQR) and *t*-test for variables represented by mean (SD), chi-square test for comparison of proportions.

### Childhood trauma and the relationship to adiposity and lipid measures

#### Total effect of childhood trauma

Experience of three or more subtypes of childhood trauma was a significant predictor (*B* = 3.145; *t* = 2.459; *p* = 0.014 [*p* = 0.011 adjusting for AP use previous 5 years]) of waist circumference, but not experience of one or two subtypes of trauma (*p* = 0.74). Analyzing only the abuse dimension, no trauma variables reached significance in predicting waist circumference. There were no other significant associations between any of the trauma variables and BMI or lipid measurements in the total sample. In subanalyses of SCZ and BD, experience of three or more subtypes of childhood trauma was a significant predictor (*B* = 5.087; *t* = 2.737; *p* = 0.006) of waist circumference in the SCZ group. See Supplementary Tables 1–3 for details of the total effect model.

### Mediation effects in subsamples

#### Cognitive control

In the adjusted mediation analyses in the total sample, cognitive control was a close to significant mediator between experience of three or more subtypes of trauma and waist circumference (see Table 2, lower limit confidence interval close to zero). Moreover, cognitive control was a significant mediator between experience of one or two subtypes of childhood trauma and waist circumference. There was a significant direct effect of three or more subtypes of trauma on waist circumference (*p* = 0.021), but not for one or two subtypes of trauma (*p* = 0.60). In subanalyses of SCZ and BD, cognitive control was a close to significant mediator between experience of three or more subtypes of trauma and waist circumference (lower limit confidence interval close to zero; direct effect, *p* = 0.016) and a significant mediator between experience of one or two subtypes of childhood trauma and waist circumference (direct effect, *p* = 0.50) in the SCZ group (see Supplementary Table 4).

**Table 2.** Cognitive and personality characteristics in mediation analyses of CTQ and waist circumference.

Waist circumference					
IV^a^	Mediator^b^	Indirect effect	SE	95% CI	
				Lower	Upper
CTQ, *p* < 0.001	Cognitive control, *p* < 0.001	X1: 0.3160^c^	0.1887	0.0079	0.7400
		X2: 0.2505	0.1844	−0.0164	0.6896
	Impulsiveness, *p* < 0.001	X1: 0.0849	0.2942	−0.4792	0.7147
		X2: 0.2251	0.7633	−1.1685	1.8033
	Self-esteem, *p* < 0.001	X1: 0.1131	0.2868	−0.4346	0.6889
		X2: 0.1755	0.4439	−0.6757	1.0768
	Affective lability, *p* < 0.001	X1: −0.0400	0.4036	−0.8230	0.8330
		X2: −0.0553	0.5473	−1.1348	1.1563

a *p* value based on the following model (*N* = 743): CTQ (0 vs. 1–2 vs. 3 or more subtypes of trauma), age, sex, metabolic propensity of antipsychotic drug, diagnostic group (IVs); Waist circumference (DV).

b *p* value based on the following model in subsamples with mediator: CTQ (0 vs. 1–2 vs. 3 or more subtypes of trauma), mediator, age, sex, metabolic propensity of antipsychotic drug, diagnostic group (IVs); waist circumference (DV).

c Significant indirect effect.

X1: One or two subtypes of trauma relative to no trauma.

X2: Three or more subtypes of trauma relative to no trauma.

Abbreviations: CI, confidence interval; CTQ, Childhood Trauma Questionnaire; DV, dependent variable; IV, independent variable; SE, standard error.

#### Impulsiveness, self-esteem, and affective lability

Impulsiveness measured by BIS-11, self-esteem measured by RSES, and affective lability measured by ALS-18 were not significant mediators between childhood trauma and waist circumference.

See Table 2 for an overview of cognitive and personality characteristics in mediation analyses of childhood trauma and waist circumference in the total sample.

## Discussion

The current study found an association between experience of three or more subtypes of childhood trauma and waist circumference in patients with SMD. Furthermore, cognitive control was identified as a significant mediator between exposure to one or two subtypes of childhood trauma and waist circumference and a close to significant mediator of three or more subtypes of childhood trauma. Subgroup analyses indicated associations mainly to be of importance in SCZ. The findings suggest that diminished cognitive control could be a sequela of early trauma affecting the regulation of health behavior, with increased risk of central obesity.

In line with the current results, positive associations between childhood trauma and cardiometabolic risk factors have been shown for the general population [20,73]. A review found child maltreatment to be associated with CVD in 91.7% of studies [74]. Furthermore, a study indicated early trauma as a significant predictor of adult obesity independent of having a psychiatric diagnosis [75]. In SMDs, there is a sparsity of studies, and conclusions are hampered by less rigid adjustments of medication effects, smaller sample sizes, and conflicting results. Consistent with our findings, some studies have indicated a relationship between childhood adversities and adiposity measures [22,23]. However, Misiak et al. [25] reported that individuals with first-episode schizophrenia with and without a history of childhood trauma did not differ significantly in BMI and that there was no significant association between number of childhood adversities and BMI. Discrepancies to our results might be due to different participation criteria as our study also included bipolar disorder and chronic illness conditions, and Misiak et al. [25] excluded patients with substance use issues. Moreover, the current sample is several times larger, suggesting more robust findings. The findings are also in line with studies of other patient groups. In a survey concerning self-reported adverse childhood experiences, high adversity was positively associated with CVD in individuals with depression [76]. A cohort study of individuals with anxiety and depression as well as healthy controls found an increased overall CVD risk in individuals with a history of sexual abuse, and that both psychological as well as sexual abuse was related to higher waist circumference [51].

No significant associations were found between experience of childhood trauma and any of the other cardiometabolic risk factors, that is, BMI and lipid measures. However, waist circumference is more specific for central obesity than BMI and is not a strong predictor of lipid levels [77]. In an earlier study from our group with a partly overlapping sample, Aas et al. [27] reported an association between severity of childhood abuse and BMI. In comparison, the current sample has been increased with several hundred individuals and there is a close correlation between BMI and waist. However, one might speculate whether early trauma is more closely linked to abdominal adiposity than other adiposity patterns, not readily detected by the BMI measure, and with little clinically relevant effects on serum lipids. Importantly, waist circumference is a central part of CVD risk assessment and is closely linked to morbidity and mortality [78]. Lack of significant associations with lipids might be related to inter-individual variation of the effect of diet on lipids, which may partly be based on genetic variation [79].

In the current study, the group with the highest number of experiences of subtypes of childhood trauma had significantly increased waist circumference in the analyses of total effect. Although this level of exposure was involved in a close to significant mediation, one or two subtypes of trauma were the basis for the significant mediator pathway of cognitive control. Mediating effects can be detected despite a nonsignificant main effect [72], thus together the results seem to suggest early trauma as a risk factor for obesity with impairments in cognitive control as a mediating mechanism. However, other mechanisms might be of increasing importance with higher levels of trauma. Interestingly, childhood adversities may lead to disturbances in bodily regulation systems altering immune, neuroendocrine, and autonomic functioning [80,81]. More extensive trauma experiences could be more closely related to such biological components [82,83].

The mediation analyses indicate cognitive control as an underlying factor of behavior linking childhood trauma and weight gain. Children exposed to early adversity have a heightened risk of being emotionally reactive to stress, in addition to the risk of being less capable of regulating emotions [84]. Several studies indicate that individuals with experiences of childhood trauma may be more prone to develop maladaptive coping strategies like emotional eating [85,86]. In an early study by Felitti et al. [85], it was indicated that children experiencing adversity have a heightened risk of maladaptive coping mechanisms like smoking and overeating in response to high levels of distress. Moreover, survivors of severe childhood adversities like violence seem prone to consume larger quantities of food and eat in response to stress in addition to experiencing higher levels of anger, stress, and depressed mood [28,87]. Studies have found that different forms of childhood adversities often co-occur [85] and that exposure to several subtypes of trauma is associated with having more complex trauma symptoms [88]. Based on the current findings, it is possible that an upbringing characterized by adverse experiences, such as lack of support and psychological nourishment, may lead to suppression of feelings through eating as a maladaptive coping strategy related to cognitive impairments [85,89]. Interestingly, executive dysfunction and emotional control seem to be inversely associated [90], indicating the effects of cognitive control in emotional regulation. Adding to this, findings indicate that executive dysfunction, such as reduced behavioral inhibition, is associated with negative eating behaviors, including intake of palatable foods and obesity [91]. The mechanism might include impaired suppression of memories and clues related to food and eating, leading to unfavorable eating behavior [92]. These patterns are of great interest given the high frequency of experience of childhood trauma in SMDs [93]. Compared to personality characteristics [94,95], the findings suggest a more prominent role of cognitive impairments in development of central obesity in traumatized individuals with SMD.

The main strength of the study is the well-characterized sample enabling adjustments of several potential confounding factors in both the main analyses as well as in the mediation analyses. Several personality characteristics were measured and investigated by use of thoroughly validated questionnaires, and the measure cognitive control was obtained from a rigorous neuropsychological assessment. Importantly, the study highlights the value of well-controlled analyses including metabolic side-effects of antipsychotic agent [71] as well as age and sex, as these variables were significant predictors of cardiometabolic risk across analyses. The sample size allowed for subanalyses, suggesting effects to be mainly of relevance in the SCZ group.

There are some limitations that warrant mentioning. There is a risk for false-positive results due to the number of analyses performed. We adjusted the significance level, however, only to a moderate extent, allowing potential interesting associations to be detected. Despite substantial sample sizes, small effects may go unnoticed; thus limitations in sample sizes, especially in the analyses of the different personality characteristics, might be a cause of negative findings. Although analyses were adjusted for AP propensity of metabolic side-effects, AP dosage cannot be ruled out as a confounder. However, this seems less likely given the limited support of correlation between dosage and weight gain of the majority of APs [96]. Moreover, data on childhood trauma were collected retrospectively and low consistency has been indicated between childhood maltreatment measured by prospective and retrospective measures [97,98]. However, both prospective and retrospective measures are predictive of later adolescent outcomes [99,100]. Age at traumatization was not recorded, meaning we could not test effects at specific age groups. Moreover, the current study lacks physiological and neural data that could indicate biological mechanisms of the suggested associations. Lastly, we cannot make inferences about causality based on the cross-sectional design. For instance, we do not know the sequence of the events, thus obesity might have occurred before exposure to childhood trauma.

In summary, the present study suggests a relationship between childhood trauma and waist circumference mediated by cognitive control in patients with SMDs. Adverse health behavior based on diminished cognitive control associated with early trauma might thus be a factor in central obesity in adult age. The suggested associations are important for this group of patients given their high level of experienced childhood trauma. Clinical implications include an increased focus on childhood trauma and emphasis on cognitive regulatory mechanisms to improve health behavior. Ultimately, interventions targeting these factors may contribute to more positive long-term physical health outcomes as well as prolonged life expectancy for patients with SMDs.

## Data Availability

The data that support the findings of this study will be made available upon request.

## Supplementary material

For supplementary material accompanying this paper visit http://dx.doi.org/10.1192/j.eurpsy.2021.14. click here to view supplementary material
