# Supplementary material for: Childhood trauma and cardiometabolic risk in severe mental disorders: The mediating role of cognitive control
Source: Eur Psychiatry. 2021 Mar 29;64(1):e24. doi: 10.1192/j.eurpsy.2021.14 (PMC8084596; doi:10.1192/j.eurpsy.2021.14)
Supplement: Supplementary file 1 [file epasup.zip › S0924933821000146sup004.docx]

Supplementary Table 4

Cognitive and personality characteristics in mediation analyses of childhood trauma (CTQ) and waist circumference in SCZ and BD

| **Waist circumference** | | | | | |
| --- | --- | --- | --- | --- | --- |
|  | **Mediator**^b^ | **Indirect effect** | **SE** | **95% CI** | |
|  |  |  |  | **Lower** | **Upper** |
| ***SCZ*** |  |  |  |  |  |
| CTQ^a^ (IV), p<0.001 | Cognitive control, p<0.001 | X1: 0.4506^*^ | 0.2740 | 0.0340 | 1.0971 |
|  |  | X2: 0.5154 | 0.3858 | -0.0920 | 1.4080 |
|  | Impulsiveness, p<0.001 | X1: -0.0150 | 0.4401 | -0.9948 | 0.8875 |
|  |  | X2: -0.0422 | 1.1490 | -2.3093 | 2.3413 |
|  | Self-esteem, p<0.001 | X1: -0.0492 | 0.3314 | -0.7208 | 0.6392 |
|  |  | X2: -0.0983 | 0.6438 | -1.3603 | 1.2212 |
|  | Affective lability, p=0.0013 | X1: -0.1335 | 0.9766 | -2.0991 | 2.0283 |
|  |  | X2: -0.2378 | 1.5245 | -3.0420 | 3.1531 |
| ***BD*** |  |  |  |  |  |
| CTQ^a^ (IV), p<0.001 | Cognitive control, p<0.001 | X1: 0.0443 | 0.2428 | -0.3972 | 0.5908 |
|  |  | X2: 0.0205 | 0.1469 | -0.2850 | 0.3506 |
|  | Impulsiveness, p<0.001 | X1: 0.0782 | 0.3950 | -0.7779 | 0.9035 |
|  |  | X2: 0.2244 | 1.0065 | -1.6049 | 2.4881 |
|  | Self-esteem, p<0.001 | X1: 0.4053 | 0.5132 | -0.5625 | 1.5083 |
|  |  | X2: 0.4838 | 0.6109 | -0.7138 | 1.7629 |
|  | Affective lability, p<0.001 | X1:-0.0422 | 0.5071 | -1.0746 | 1.0735 |
|  |  | X2:-0.0449 | 0.5476 | -1.1348 | 1.2234 |

^a^p-value based on following model (SCZ N=406; BD N=337): CTQ [0 vs. 1-2 vs. 3 or more subtypes of trauma], age, sex, metabolic propensity of antipsychotic drug (IVs); Waist circumference (DV).

^b^p-value based on following model in subsamples with mediator: CTQ [0 vs. 1-2 vs. 3 or more subtypes of trauma], mediator, age, sex, metabolic propensity of antipsychotic drug (IVs); Waist circumference (DV).

^*^Significant indirect effect.

X1: One or two subtypes of trauma relative to no trauma.

X2: Three or more subtypes of trauma relative to no trauma.

Abbreviations: BD= Bipolar Spectrum Disorders; CI= Confidence Interval; CTQ= Childhood Trauma Questionnaire; DV= Dependent Variable; IV= Independent Variable; Schizophrenia= Schizophrenia Spectrum Disorders; SE = Standard Error.
