# Supplementary material for: Childhood trauma and cardiometabolic risk in severe mental disorders: The mediating role of cognitive control
Source: Eur Psychiatry. 2021 Mar 29;64(1):e24. doi: 10.1192/j.eurpsy.2021.14 (PMC8084596; doi:10.1192/j.eurpsy.2021.14)
Supplement: Supplementary file 1 [file epasup.zip › S0924933821000146sup003.docx]

Supplementary Table 3

Childhood trauma and the relationship to adiposity and lipid measures in BD, total effect model

|  | B | t | p-value |
| --- | --- | --- | --- |
| *Waist circumference* |  |  |  |
| 1 or 2 subtypes of trauma | 0.349 | 0.239 | 0.811 |
| ≥ 3 subtypes of trauma | 1.153 | 0.671 | 0.503 |
| Age | 0.365 | 6.590 | <0.001 |
| Sex | -9.318 | -7.278 | <0.001 |
| AP metabolic side effect, high | 4.269 | 3.135 | 0.002 |
| AP metabolic side effect, low | 2.197 | 1.082 | 0.280 |
| *BMI* |  |  |  |
| 1 or 2 subtypes of trauma | -0.001 | -0.067 | 0.947 |
| ≥ 3 subtypes of trauma | 0.004 | 0.168 | 0.867 |
| Age | 0.003 | 4.373 | <0.001 |
| Sex | -0.038 | -2.101 | 0.036 |
| AP metabolic side effect, high | 0.044 | 2.281 | 0.023 |
| AP metabolic side effect, low | 0.043 | 1.516 | 0.130 |
| *Total Cholesterol* |  |  |  |
| 1 or 2 subtypes of trauma | 0.026 | 0.213 | 0.831 |
| ≥ 3 subtypes of trauma | -0.066 | -0.437 | 0.662 |
| Age | 0.025 | 5.151 | <0.001 |
| Sex | -0.065 | -0.589 | 0.556 |
| AP metabolic side effect, high | 0.200 | 1.710 | 0.088 |
| AP metabolic side effect, low | 0.365 | 2.038 | 0.042 |
| *HDL-Cholesterol* |  |  |  |
| 1 or 2 subtypes of trauma | 0.063 | 1.170 | 0.243 |
| ≥ 3 subtypes of trauma | -0.014 | -0.211 | 0.833 |
| Age | 0.002 | 0.879 | 0.380 |
| Sex | 0.304 | 6.418 | <0.001 |
| AP metabolic side effect, high | -0.092 | -1.832 | 0.068 |
| AP metabolic side effect, low | -0.071 | -0.924 | 0.356 |
| *LDL-Cholesterol* |  |  |  |
| 1 or 2 subtypes of trauma | 0.015 | 0.147 | 0.883 |
| ≥ 3 subtypes of trauma | -0.002 | -0.016 | 0.978 |
| Age | 0.016 | 4.123 | <0.001 |
| Sex | -0.154 | -1.674 | 0.095 |
| AP metabolic side effect, high | 0.263 | 2.692 | 0.008 |
| AP metabolic side effect, low | 0.353 | 2.309 | 0.022 |
| *Triglycerides* |  |  |  |
| 1 or 2 subtypes of trauma | -0.018 | -0.271 | 0.787 |
| ≥ 3 subtypes of trauma | -0.139 | -1.700 | 0.090 |
| Age | 0.006 | 2.233 | 0.026 |
| Sex | -0.258 | -4.341 | <0.001 |
| AP metabolic side effect, high | 0.154 | 2.429 | 0.016 |
| AP metabolic side effect, low | 0.138 | 1.430 | 0.154 |

Abbreviations: AP metabolic side effect= Antipsychotic agent propensity of metabolic side effect; BD= Bipolar spectrum disorder; HDL-Cholesterol= high-density lipoprotein-Cholesterol; LDL-Cholesterol= low-density lipoprotein-Cholesterol; 1 or 2 subtypes of trauma or ≥3 subtypes of trauma= Meeting the moderate to severe cut-off score for 1 or 2 subtype(s) or 3 or more subtypes of childhood trauma, respectively, based on the Childhood Trauma Questionnaire (CTQ).
