# Supplementary material for: Childhood trauma and cardiometabolic risk in severe mental disorders: The mediating role of cognitive control
Source: Eur Psychiatry. 2021 Mar 29;64(1):e24. doi: 10.1192/j.eurpsy.2021.14 (PMC8084596; doi:10.1192/j.eurpsy.2021.14)
Supplement: Supplementary file 1 [file epasup.zip › S0924933821000146sup002.docx]

Supplementary Table 2

Childhood trauma and the relationship to adiposity and lipid measures in SCZ, total effect model

|  | B | t | p-value |
| --- | --- | --- | --- |
| *Waist circumference* |  |  |  |
| 1 or 2 subtypes of trauma | -0.801 | -0.550 | 0.583 |
| ≥ 3 subtypes of trauma | 5.087 | 2.737 | 0.006 |
| Age | 0.629 | 9.020 | <0.001 |
| Sex | -10.056 | -7.427 | <0.001 |
| AP metabolic side effect, high | 4.086 | 2.290 | 0.023 |
| AP metabolic side effect, low | 3.191 | 1.706 | 0.089 |
| *BMI* |  |  |  |
| 1 or 2 subtypes of trauma | -0.021 | -1.007 | 0.314 |
| ≥ 3 subtypes of trauma | 0.041 | 1.521 | 0.129 |
| Age | 0.006 | 6.058 | <0.001 |
| Sex | -0.048 | -2.513 | 0.012 |
| AP metabolic side effect, high | 0.045 | 1.766 | 0.078 |
| AP metabolic side effect, low | 0.040 | 1.483 | 0.139 |
| *Total Cholesterol* |  |  |  |
| 1 or 2 subtypes of trauma | 0.231 | 1.995 | 0.047 |
| ≥ 3 subtypes of trauma | 0.301 | 2.112 | 0.035 |
| Age | 0.029 | 5.132 | <0.001 |
| Sex | -0.167 | -1.551 | 0.122 |
| AP metabolic side effect, high | 0.100 | 0.699 | 0.485 |
| AP metabolic side effect, low | 0.083 | 0.556 | 0.578 |
| *HDL-Cholesterol* |  |  |  |
| 1 or 2 subtypes of trauma | -0.003 | -0.068 | 0.946 |
| ≥ 3 subtypes of trauma | -0.047 | -0.827 | 0.409 |
| Age | 0.005 | 2.091 | 0.037 |
| Sex | 0.301 | 7.009 | <0.001 |
| AP metabolic side effect, high | -0.159 | -2.784 | 0.006 |
| AP metabolic side effect, low | -0.140 | -2.338 | 0.020 |
| *LDL-Cholesterol* |  |  |  |
| 1 or 2 subtypes of trauma | 0.147 | 1.402 | 0.162 |
| ≥ 3 subtypes of trauma | 0.230 | 1.771 | 0.077 |
| Age | 0.019 | 3.709 | <0.001 |
| Sex | -0.332 | -3.412 | 0.001 |
| AP metabolic side effect, high | 0.225 | 1.725 | 0.085 |
| AP metabolic side effect, low | 0.137 | 1.004 | 0.316 |
| *Triglycerides* |  |  |  |
| 1 or 2 subtypes of trauma | 0.024 | 0.379 | 0.705 |
| ≥ 3 subtypes of trauma | 0.139 | 1.798 | 0.073 |
| Age | 0.008 | 2.606 | 0.010 |
| Sex | -0.286 | -4.904 | <0.001 |
| AP metabolic side effect, high | 0.088 | 1.129 | 0.260 |
| AP metabolic side effect, low | 0.106 | 1.307 | 0.192 |

Abbreviations: AP metabolic side effect= Antipsychotic agent propensity of metabolic side effect; HDL-Cholesterol= high-density lipoprotein-Cholesterol; LDL-Cholesterol= low-density lipoprotein-Cholesterol; 1 or 2 subtypes of trauma or ≥3 subtypes of trauma= Meeting the moderate to severe cut-off score for 1 or 2 subtype(s) or 3 or more subtypes of childhood trauma, respectively, based on the Childhood Trauma Questionnaire (CTQ); SCZ= Schizophrenia spectrum disorder.
