# Supplementary material for: Childhood trauma and cardiometabolic risk in severe mental disorders: The mediating role of cognitive control
Source: Eur Psychiatry. 2021 Mar 29;64(1):e24. doi: 10.1192/j.eurpsy.2021.14 (PMC8084596; doi:10.1192/j.eurpsy.2021.14)
Supplement: Supplementary file 1 [file epasup.zip › S0924933821000146sup001.docx]

Supplementary Table 1

Childhood trauma and the relationship to adiposity and lipid measures, total effect model

|  | B | t | p-value |
| --- | --- | --- | --- |
| *Waist circumference* |  |  |  |
| 1 or 2 subtypes of trauma | -0.343 | -0.330 | 0.741 |
| ≥ 3 subtypes of trauma | 3.145 | 2.459 | 0.014 |
| Age | 0.482 | 10.870 | <0.001 |
| Sex | -9.409 | -10.022 | <0.001 |
| Diagnostic group | 1.184 | 1.184 | 0.237 |
| AP metabolic side effect, high | 4.150 | 3.764 | <0.001 |
| AP metabolic side effect, low | 2.761 | 2.101 | 0.036 |
| *BMI* |  |  |  |
| 1 or 2 subtypes of trauma | -0.013 | -0.861 | 0.389 |
| ≥ 3 subtypes of trauma | 0.024 | 1.281 | 0.201 |
| Age | 0.005 | 7.304 | <0.001 |
| Sex | -0.041 | -3.104 | 0.002 |
| Diagnostic group | 0.007 | 0.484 | 0.629 |
| AP metabolic side effect, high | 0.044 | 2.822 | 0.005 |
| AP metabolic side effect, low | 0.040 | 2.125 | 0.034 |
| *Total Cholesterol* |  |  |  |
| 1 or 2 subtypes of trauma | 0.146 | 1.719 | 0.086 |
| ≥ 3 subtypes of trauma | 0.142 | 1.372 | 0.170 |
| Age | 0.026 | 7.153 | <0.001 |
| Sex | -0.114 | -1.487 | 0.137 |
| Diagnostic group | 0.193 | 2.364 | 0.018 |
| AP metabolic side effect, high | 0.176 | 1.944 | 0.052 |
| AP metabolic side effect, low | 0.183 | 1.690 | 0.091 |
| *HDL-Cholesterol* |  |  |  |
| 1 or 2 subtypes of trauma | 0.025 | 0.724 | 0.469 |
| ≥ 3 subtypes of trauma | -0.031 | -0.726 | 0.468 |
| Age | 0.003 | 2.043 | 0.041 |
| Sex | 0.306 | 9.714 | <0.001 |
| Diagnostic group | -0.029 | -0.860 | 0.390 |
| AP metabolic side effect, high | -0.121 | -3.247 | 0.001 |
| AP metabolic side effect, low | -0.103 | -2.303 | 0.022 |
| *LDL-Cholesterol* |  |  |  |
| 1 or 2 subtypes of trauma | 0.098 | 1.321 | 0.187 |
| ≥ 3 subtypes of trauma | 0.135 | 1.483 | 0.139 |
| Age | 0.017 | 5.421 | <0.001 |
| Sex | -0.247 | -3.680 | <0.001 |
| Diagnostic group | 0.188 | 2.604 | 0.009 |
| AP metabolic side effect, high | 0.251 | 3.153 | 0.002 |
| AP metabolic side effect, low | 0.182 | 1.941 | 0.053 |
| *Triglycerides* |  |  |  |
| 1 or 2 subtypes of trauma | -0.004 | -0.083 | 0.934 |
| ≥ 3 subtypes of trauma | 0.016 | 0.284 | 0.776 |
| Age | 0.007 | 3.392 | 0.001 |
| Sex | -0.265 | -6.398 | <0.001 |
| Diagnostic group | 0.036 | 0.812 | 0.417 |
| AP metabolic side effect, high | 0.128 | 2.610 | 0.009 |
| AP metabolic side effect, low | 0.127 | 2.167 | 0.031 |

Abbreviations: AP metabolic side effect= Antipsychotic agent propensity of metabolic side effect; Diagnostic group= Schizophrenia spectrum disorder, Bipolar spectrum disorder; HDL-Cholesterol= high-density lipoprotein-Cholesterol; LDL-Cholesterol= low-density lipoprotein-Cholesterol; 1 or 2 subtypes of trauma or ≥3 subtypes of trauma= Meeting the moderate to severe cut-off score for 1 or 2 subtype(s) or 3 or more subtypes of childhood trauma, respectively, based on the Childhood Trauma Questionnaire (CTQ).
